# A Valuable Product of Microbial Cell Factories: Microbial Lipase

**DOI:** 10.3389/fmicb.2021.743377

**Published:** 2021-09-20

**Authors:** Wentao Yao, Kaiquan Liu, Hongling Liu, Yi Jiang, Ruiming Wang, Wei Wang, Tengfei Wang

**Affiliations:** ^1^State Key Laboratory of Biobased Material and Green Papermaking (LBMP), Qilu University of Technology (Shandong Academy of Sciences), Jinan, China; ^2^Key Laboratory of Shandong Microbial Engineering, College of Bioengineering, QiLu University of Technology (Shandong Academy of Sciences), Jinan, China; ^3^State Key Laboratory of Microbial Metabolism, School of Life Sciences and Biotechnology, Shanghai Jiao Tong University, Shanghai, China

**Keywords:** lipase, microbial source, heterologous expression, industry application, future perspectives

## Abstract

As a powerful factory, microbial cells produce a variety of enzymes, such as lipase. Lipase has a wide range of actions and participates in multiple reactions, and they can catalyze the hydrolysis of triacylglycerol into its component free fatty acids and glycerol backbone. Lipase exists widely in nature, most prominently in plants, animals and microorganisms, among which microorganisms are the most important source of lipase. Microbial lipases have been adapted for numerous industrial applications due to their substrate specificity, heterogeneous patterns of expression and versatility (i.e., capacity to catalyze reactions at the extremes of pH and temperature as well as in the presence of metal ions and organic solvents). Now they have been introduced into applications involving the production and processing of food, pharmaceutics, paper making, detergents, biodiesel fuels, and so on. In this mini-review, we will focus on the most up-to-date research on microbial lipases and their commercial and industrial applications. We will also discuss and predict future applications of these important technologies.

## Introduction

Lipases (EC 3.1.1.3), also known as triacylglycerol acyl hydrolase, are serine hydrolases that catalyze the removal of esterified fatty acids from complex compounds (Chandra et al., 2020). Lipases are important industrial enzymes that are used widely in various production strategies. Lipases are the third most commonly used enzyme class after proteases and amylases (Ulker et al., 2011). It could catalyze the hydrolysis of various forms of long-chain triacylglycerides to form free fatty acids and glycerol, and the synthesis of fatty acid glycerides based on fatty acids and glycerol, which is a class of carboxylic ester hydrolases (EC 3.1.1.X) (Geoffry and Achur, 2017).

Lipases are divided into three categories based on their location and substrate specificity; these include non-specific lipases, 1, 3-specific lipases, and fatty-acid specific lipases. Non-specific lipases catalyze the hydrolysis of triglycerides into free fatty acids and glycerol via monoglyceride and diglyceride intermediates; these enzymes are capable of removing fatty acids from any of the three esterified sites of the original substrate, although hydrolysis of the monoglyceride and diglyceride intermediates proceeds more rapidly than that of the original triglyceride substrate (Ferreira-Dias et al., 2013). The 1, 3-specific lipases catalyze the release of fatty acids from the 1 and 3 carbon sites of the of triglyceride substrate; they are not capable of performing hydrolysis at the middle, or second ester bond. The 1, 3-specific lipases promote hydrolysis of triglycerides into monoglycerides at a more rapid rate than performed by non-specific lipases (Kavadia et al., 2018; López-Fernández et al., 2019; Gao et al., 2020). By contrast, fatty-acid-specific lipases demonstrate substrate selectivity; these enzymes target long-chain fatty acids with *cis*-double bonds between C-9 and C-10 (Kapoor and Gupta, 2012).

**FIGURE 1:**
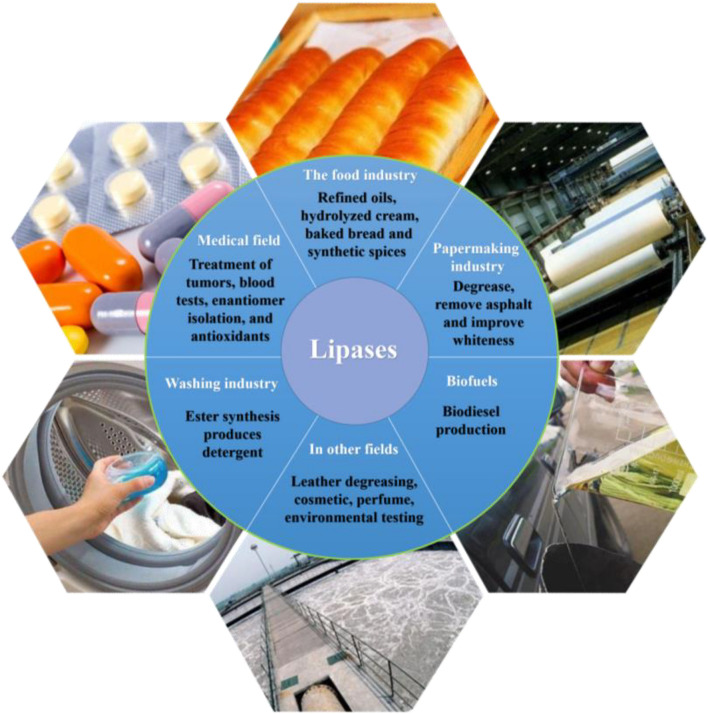
Various industrial applications that feature microbial lipases.

Lipases that catalyze the hydrolysis of triacylglycerol into fatty acids and glycerol, as well as into monoacylglycerol and diacylglycerol can also be employed to promote both esterification and transesterification reactions in both organic or inorganic solvents (Mouad et al., 2016; Jiao et al., 2018; Avhad and Marchetti, 2019). Among the properties of this environmentally friendly, or “green” catalyst, lipase has high catalytic efficiency, can function under comparatively mild reaction conditions, involves no coenzymes or other cofactors or by-products; as such, it is in wide use in various industrial fields including food, pharmaceutical, paper, detergent, and biodiesel production (Figure 1; Borowiecki et al., 2017; Dumorne et al., 2017; Sadaf et al., 2018; Vanleeuw et al., 2019).

With the development of genetic engineering technologies, more in-depth and comprehensive research reports have been published that focus on lipase gene expression, protein structure and function as well as its mechanism of action at the molecular level. Application of molecular cloning technologies facilitated the generation of systems for heterologous expression of microbial-derived lipases in *Escherichia coli*, *Bacillus subtilis*, *Pichia pastoris*, and *Saccharomyces cerevisiae*. Current research efforts focus on the screening of various lipase strains, the modification of existing lipase genes, analysis of modified lipase function, and the broader applicability of lipases in industrial settings.

This review discusses the research status of microbial lipase, discusses the main sources and expression of microbial lipase, lists the effects of physical and chemical factors on microbial lipase, and summarizes the latest research on microbial lipase and its commercial and industrial applications.

## Source of Microbial Lipases

The earliest lipase studied (in the 1830s) was an enzyme preparation isolated from rabbit pancreas; interestingly, plant-derived lipase was not discovered until the 1970s. Lipases derived from microbial sources were first identified early in the 20th century (Jaeger et al., 1994). Until recent years, metagenomics lipases and artificially designed lipases have gradually appeared in people’s vision (Tang et al., 2017; Cen et al., 2019; Almeida et al., 2020; Verma et al., 2021).

Lipases can be identified in many forms and variants in animals, plants and microorganisms (Sarmah et al., 2017); the microbial lipases are notably diverse in nature (Gupta et al., 2004). Lipase activity is of central importance in all living organisms, as it modulates critical physiologic processes involved in digestion and absorption, as well as fat and lipoprotein metabolism (Winkler et al., 1990).

Compared with animals and plants, microorganisms encompass a wide variety of species, undergo rapid growth and reproduction, are typically easy to manipulate experimentally, and can be associated with significant genetic variation. Microbial lipases have received extensive attention in industrial applications due to its selectivity, stability and wide substrate specificity (Kumar et al., 2016). Furthermore, lipases produced by some microorganisms that live in extreme habitats have unique properties that are often more conducive for industrial production (Verma et al., 2021). Most lipases identified in microorganisms are secretory extracellular enzymes; they can be isolated at high purity and are thus suitable for mass production. As such, microbial lipases are the main source of industrial enzymes and present important value as well with respect to theoretical research.

Among microorganisms, lipase is most abundant in bacteria, fungi, and yeast. The most common bacterial sources for lipases include *Bacillus* spp., *Pseudomonas* spp., *Staphylococcus* sp., and *Burkholderia* sp. (Bharathi and Rajalakshmi, 2019; Priyanka et al., 2019). Bacterial lipase catalyzed the most types of hydrolytic reactions and the highest levels of activity; the bacterial enzymes were also among the most stable. Of this group, the lipase isolated from *Pseudomonas* spp. had the overall best performance (Panizza et al., 2013). Fungal lipases have been used extensively in a variety of biotechnology applications due to their stability, specificity, and ease of production. Among these, lipases isolated from *Thermomyces lanuginosus*, *Rhizopus oryzae*, and *Aspergillus niger* all have important industrial value. In yeast, lipases of the genus *Candida* have been used for production and also for research; among these are lipases isolated from *Candida antarctica* and *Candida rugosa* (Sarmah et al., 2017).

## Identification of Lipase Activity in Microorganisms and Analysis of Its Enzymatic Properties

### Determination of Lipase Activity

Many methods have been used to identify lipase activity; these include both immunological and physicochemical methods.

### Immunological Methods

Immunological methods can be used to quantify lipases in biological solvents, independent of its enzymatic activity (Sarmah et al., 2018). Immunological methods used for this purpose include immunoblot (Western blot) analysis (Bezzine et al., 1998), radioimmunoassays (RIAs), and Enzyme-Linked Immunosorbent Assays (ELISAs; Miyashita et al., 2017). ELISAs are highly sensitive and specific for this purpose. Dati and Grenner detected and quantified pure lipase with this methodology (Dati and Grenner, 1984); Miyashita et al. has established a new ELISA method using newly generated monoclonal Abs (MoAbs) for quantification of serum hepatic triglyceride (TG) lipase (HTGL), as well as postheparin plasma hepatic triglyceride lipase (PHP-HTGL; Miyashita et al., 2017). Immunological methods can be time-consuming to develop given the months required for antibody generation; these methods have some limitations with respect to practical application.

### Physical and Chemical Methods

Physical and chemical methods include plate methods (Momsen and Brockman, 1997), colorimetric methods (Hiol et al., 1999), and titration methods (Singh and Mukhopadhyay, 2012). The plate assay is a rapid, qualitative detection method, that was developed in 1987 by Kouker and Jaeger using Rhodamine B as an indicator (Kouker and Jaeger, 1987). This method facilitated the detection of the bacterial lipase under UV irradiation, as substrate hydrolysis resulted in the formation of a fluorescent orange halo, with the degree and extent of the activity reflected in its diameter. Although this method is simple, it is not precisely quantitative, and as such is more commonly used for qualitative detection only.

The colorimetric method is more accurate, reliable, and sensitive, but it requires specific equipment and complicated procedures. Titration is a system that involves substrate, buffer, and enzyme solution (Dharmsthiti et al., 1998). Reactions are carried out at specific temperatures and pH; they are then terminated with 95% ethanol. After adding indicators, the reaction is titrated with a standard alkaline solution. This method is simple and generates results rapidly. Although this procedure is associated with some errors, it is still widely used in practice.

The common substrates used to detect lipase activity by physicochemical methods include triglycerides and olive oil (Sri Kaja et al., 2018). Other versions of this assay feature synthetic or semi-synthetic triglycerides, chromogenic substrates, or radioactive tracer substrates. Given the cost of trioleate glyceride, olive oil is typically used as a substrate; olive oil contains more than 70% oleic acid, is comparatively inexpensive, and is widely available (Vorderwülbecke et al., 1992).

### Enzymatic Properties of Microbial Lipases

Lipase catalyzes a variety of reactions; however, physical and chemical factors associated with these reactions can have a significant impact on enzyme activity. These factors include temperature, pH, as well as the presence of metal ions, organic solvents, and surfactants. Lipases from various microorganisms as well as different lipases within a single strain show great differences with respect to physical and chemical properties; as such, it is of critical importance and great value to identify the optimal enzyme and critical conditions for each industrial application. The properties of several characterized microbial lipases are summarized in Table 1.

**TABLE 1:** Some enzymatic properties of microbial lipase.

Lipase source	Optimal temperature	Optimal pH	Substrate specificity	Effects of ions, organic solvents, surfactants and other		References
				compounds on lipase activity		
				Promote	Inhibition	
Metagenomic library	74 °C	7.8	short-chain (*C* ≤ 6) and long-chain fatty acids (*C* ≥ 12)	Hg^2+^,1% Methanol, 1% Ethanol	Cu^2+^,Fe^2+^,Ca^2+^, Co^2+^,Mg^2+^,15% Methanol,15% Ethanol,15% Isopropanol	Tang et al., 2017
*Bacillus* F19L	10 °C	7.0	pNP-esters of shorter chain	—	—	Goomber et al., 2016
*Halocynthiibacter arcticus*	20 °C	8.0	p-nitrophenyl butyrate (p-NB, C4)	0.5–1.0 M Na^+^	10% Ethanol,Triton X-100,SDS,Urea	Le et al., 2020
*Aspergillus niger* GZUF36	35 °C	4.0	—	—	—	Xing et al., 2020
*Bacillus licheniformis* NCU CS-5	40 °C	8.0	Medium- and long-chain fatty acids (C6-C18)	Mg^2+^,K^+^,Na^+^, Ca^2+^,Al^2+^,Fe^3+^, Glycerol,SDS,Tween-20,Tween-60,Tween-80,CTAB	Mn^2+^,Zn^2+^,Cu^2+^,Co^2+^, Sn^2+^,Methanol, Ethanol,Acetonitrile,Dimethyl sulfoxide,Acetone Ethyl acetate, Tetrahydrofuran,Pyridine,Urea, DTT,EDTA,NaF,Na_3_VO_4_,PMSF, H_2_O_2_	Zhao et al., 2021
*Burkholderia ubonensis* SL-4	65 °C	8.5	Medium long chain pNP-esters(C8-C16)	Ca^2+^,Mn^2+^,Triton X-100,Tween-60,Ethylene Glycol	Zn^2+^,Al^3+^,EDTA, SDS,PMSF,DTT, N-butanol,N-hexanol	Yang et al., 2016
*Janibacter* sp. R02	80 °C	8.0∼9.0	MUF-butyrate	Na^+^/K^+^,Combination,Urea	Mn^2+^,Co^2+^,Cu^2+^,Ni^2+^, Fe^2+^,SDS, Triton X-100	Castilla et al., 2016
*Micrococcus luteus* EMP48-D	—	5.0	olive oil	Fe^2+^,Isopropanol,Butanol, Ethanol,Acetonitrile,Methanol	K^+^,Mg^2+^,Mn^2+^,Na^+^,Ca^2+^,N-hexane,N-heptane	Adina et al., 2021
*Thermomyces lanuginosus*	60 °C	7.0	—	—	—	Facin et al., 2018
*Thermomyces lanuginosus*	60 °C	8.5	—	—	—	Kumar et al., 2019
*Rhizopus oryzae*	45 °C	9.0	—	—	—	Asmat et al., 2018
*Streptomyces bacillaris*	45 °C	9.0	p-nitrophenyl phosphate (p-NPP)	Mg^2+^,Ba^2+^, Ca^2+^ ,K^+^,Span 80	Zn^2+^,Ni^+^,EDTA,TritonX-100,Tween-80,Tween-20,SDS	Gao et al., 2021
*Aeribacillus pallidus*	65 °C	10.0	short chain triacylglycerols	Ca^2+^,Zn^2+^, Fe^2+^,Mg^2+^	EDTA,PMSF,β-mercaptoethanol, SDS,Isooctane,Hexane, chloroform,Urea, Guanidine Hydrochloride	Ktata et al., 2019
*Candida viswanathii*	45 °C	4.0	Long chain methyl ester substrate(C16)	Mn^2+^,DTT, β-mercaptoEthanol	Co^2+^,Hg^2+^,PMSF,EDTA,SDS, Tween-20, Tween-80, Sodium deoxycholate	Almeida et al., 2018
*Geobacillus stearothermophilus* AH22	50 °C	8.0∼9.0	Short chain methyl ester substrate(C4)	Co^2+^,Mn^2+^,Cu^2+^	Hg^2+^,Zn^2+^,SDS, Triton X-100	Ekinci et al., 2016
*Paenibacillus pasadenensis* CS0611	50 °C	7.0	pNP-esters (C8)	Ca^2+^,Mg^2+^,Cu^2+^, Triton X-100,Tween-80	Zn^2+^,Fe^2+^,Co^2+^,Mn^2+^,Fe^3+^, EDTA,PMSF,β-mercaptoethanol,SDS	Gao et al., 2018
*Serratia liquefaciens*	45 °C	8.0	—	Ca^2+^,Mg^2+^, Tween-20	Zn^2+^,Fe^2+^,EDTA, SDS	Han et al., 2017
*Pseudomonas aeruginosa* ES3	40 °C	9.0∼10.0	—	Ca^2+^,sodium cholate	Cd^2+^,Cu^2+^,Iodoacetic acid,PMSF	Sarac et al., 2015
*Bacillus halodurans*	60 °C	9.0	mid chain length pNP acyl esters	Cd^2+^,Ni^2+^,Fe^3+^, pCMBA,DTNB	Ba^2+^,Fe^2+^,Pb^2+^,DEPC,EDAC, PMSF,WRK	Dua and Gupta, 2017
*Rhizomucor miehei* NRRL5282	40 °C	7.0	Medium long chain pNP-esters	Mg^2+^	Hg^2+^,NBS,SDS, Hexanol,Butanol	Takó et al., 2017
*Pseudomonas moraviensis* M9	65 °C	8.0	Medium long chain pNP-esters(C8-C16)	Ca^2+^,Sr^2+^,Mn^2+^, Ba^2+^	Cu^2+^,Zn^2+^,Co^2+^,Ni^2+^,EDTA,Glycerin, PEG-400,PEG-600	Yang et al., 2015a
*Pseudomonas* sp. R0-14	60 °C	8.5	Medium and short chain long pNP-esters	Ca^2+^,Sr^2+^,Ba^2+^	Cu^2+^,Zn^2+^,Mg^2+^,Ni^2+^,EDTA, Ethyl Alcohol, Glycerin,DMSO	Yang et al., 2015b
*Pseudomonas protegens* Pf-5	55 °C	9.5	Medium and short chain long pNP-esters	Mg^2+^,Ca^2+^,Mn^2+^, Ba^2+^,K^+^,Na^+^	Fe^3+^,Zn^2+^,Cu^2+^, EDTA	Zha et al., 2014
*Thermomyces Lanuginosus*	—	—	Short-stranded pNP-esters	—	—	Xin et al., 2017
*Geobacillus kaustophilus* DSM 7263^*T*^	50 °C	8.0	short chain fatty acid (C4)	Ca^2+^	Mg^2+^,Na^+^,Zn^2+^,PMSF,DTT,β-ME,SDS, DMSO,Ethanol, 2-Propanol	Özdemir et al., 2021

### Optimal Catalytic Temperatures for Microbial Lipases

Temperature is an important parameter when determining enzyme activity. Various lipases exhibit different thermal stabilities due to structural diversity. For example, a psychrophilic lipase from *Bacillus* F19L strain exhibited peak enzyme activity at 10 °C; it maintained 80% of its peak when the temperature shifted to 5 (Goomber et al., 2016). Similarly, a lipase derived from *Halocynthiibacter arcticus* displayed a temperature optimum of 20 °C. And, HaSGNH1 exhibited high relative activities at low temperatures, retaining ∼ 70% of its maximum activity even at 0 °C (Le et al., 2020). A sn-1,3 extracellular lipase derived from *A. niger* GZUF36 has an optimal reaction temperature of 35 °C and the enzyme activity dropped when the reaction temperature was beyond 50 °C (Xing et al., 2020). Similarly, a lipase derived from *Bacillus licheniformis* NCU CS-5, has an optimal temperature at 40 °C; its activity is relatively stable when the temperature is maintained between 5 and 55 °C (Zhao et al., 2021). An interesting lipase was isolated from *T. lanuginosus* that is both heat and organic solvent resistant (lipase NS-40116). The optimal temperature for this lipase is 60 °C; enzyme maintained at 60 °C for under 24 h retains 90% of its activity (Facin et al., 2018). A thermophilic lipase from *Burkholderia ubonensis* SL-4, has an optimal temperature at 65 °C, and maintains good stability when held at 50–55 °C (Yang et al., 2016). Likewise, a thermophilic lipase derived from *Janibacter* sp. R02 has an optimal temperature of 80 °C and maintains maximum enzyme activity between 50 and 90 °C (Castilla et al., 2016).

### Optimal pH for Microbial Lipases

The optimal pH of microbial lipase is generally neutral (pH 7.0); there is no direct relationship between optimal pH and microbial species, but specific pH does have an association with the growth environments of specific microorganisms. The appropriate pH value is of critical importance for optimal lipase activity. For different lipases, the appropriate pH value can vary greatly.

The optimum pH of a solvent-resistant lipase from *Micrococcus luteus* EMP48-D was 5.0; enzyme activity significantly decreased up to pH 6.0 and reducing continuously from pH 6.0 to pH 10.0, although was more than 85% of the initial activity of LipEMP48-D after incubation for 120 min at 40 °C (Adina et al., 2021). The heat and organic solvent-resistant lipase NS-40116 from *T. lanuginosus* displays highest activity at pH = 7.0 and maintains stability between pH 5.0–9.0 (Facin et al., 2018). Another lipase from *T. lanuginosus* exhibits an optimum pH of 8.5, although high activity is maintained within the pH range of 8.0–10.0 (Kumar et al., 2019). A lipase derived from *R. oryzae* revealed an optimum pH of 9.0 (Asmat et al., 2018), although another lipase from *Streptomyces bacillaris* exhibited an optimum pH of 9.0, and high stability between pH values of 8.0–9.0 (Gao et al., 2021). A lipase from *Aeribacillus pallidus* has an optimal pH value of 10.0, and was stable within a range of pH values from 9.0 to 11.0 at 40°C; enzyme activity was maintained at 100% after 1 h under these conditions. However, the GPL retained 80% and 71% when it is respectively incubated at pH 8, pH 7 (Ktata et al., 2019).

Interestingly, a lipase isolated from *Candida viswanathii* exhibited its highest activity at pH of 4.0; at pH 2.0–3.0, the enzyme activity was reduced to 15–40%, although 80% and 90% of its activity was maintained at pH values of 3.5 and 4.5, respectively. At pH values > 6.5, the activity decreased although a second pH maximum at 8.0 was observed (Almeida et al., 2018). However, a lipase from *A. niger* exhibited its highest activity at pH 4.0, with 90% of this activity maintained at higher pH values (Zubiolo et al., 2014).

### Effects of Metal ions on Microbial Lipases

The catalytic activity of lipase can be altered in the presence of metal ions; microbial lipases exhibit a varying tolerance to metal ions. For example, a lipase derived from *B. ubonensis* SL-4 is activated by Ca^2+^ and Mn^2+^ ions, while Mg^2+^, Co^2+^, Cu^2+^, Pb^2+^, Al^3+^ and Fe^3+^ strongly inhibit its activity (Yang et al., 2016). The organic solvent-tolerant lipase from *Geobacillus stearothermophilus* AH22 was found to be activated by Co^2+^, Cu^2+^ and Mn^2+^, while Zn^2+^ and Hg^2+^ inhibited its activity (Ekinci et al., 2016). The activity of a lipase derived from *Paenibacillus pasadenensis* CS0611 was strongly activated in Ca^2+^ and Mg^2+^, and its activity was as high as 208% and 146%, while Zn^2+^, Fe^2+^, Co^2+^, Mn^2+^ and Fe^3+^ inhibited its activity (Gao et al., 2018). Interestingly, Ca^2+^ and Mg^2+^ promote the activity of a lipase from *Serratia liquefaciens*, while Zn^2+^ and Fe^2+^ are both inhibitory factors (Han et al., 2017).

### Effects of Surfactants on Microbial Lipases

Microbial lipases are typically activated at the organic-aqueous solvent interfaces; as such, surfactants have significant influence on their activity.

For example, a lipase derived from *B. ubonensis* SL-4 was activated by Tween-20, Tween-40, Tween-60, Tween-80, Triton X-100, and cetyl trimethyl ammonium bromide (CTAB), but inhibited by ethylene diamine tetraacetic acid (EDTA), dithiothreitol (DTT), beta-mercaptoethanol (β-ME) and sodium dodecyl sulfate (SDS; Yang et al., 2016). Both sodium cholate and iodoacetic acid promote the activity of a lipase derived from *P. aeruginosa* ES3, while phenyl methane sulfonyl fluoride (PMSF) serves as an inhibitor (Sarac et al., 2015). A lipase derived from *Janibacter* sp. R02, was activated by urea, but inhibited by Triton X-100 and SDS, with 50% maximum activity observed at an SDS concentration of 0.5% (w/v); PMSF had no significant impact on the enzyme activity (Castilla et al., 2016). Finally, a lipase derived from *Bacillus halodurans* remains relatively stable in commercial detergents and is not affected by EDTA, Triton X-100 and Tween 80; however it is activated by para-Chloromethyl benzoic acid (pCMBA)and 5,5′-dithio-bis-[2-nitrobenzoic acid] (DTNB) and inhibited by diethylpyrocarbonate (DEPC), PMSF, 1-ethyl-3-(3-dimethyl aminopropyl) carbodiimide (EDAC)and Woodward’s Reagent K (WRK; Dua and Gupta, 2017).

### Effects of Organic Solvents on Microbial Lipases

Lipases can catalyze reactions in aqueous solution and in organic solvents; among the reactions carried out in the latter environment, lipases catalyze transesterification and esterase activity, including ammonolysis, and acidolysis of substrate esters. Various organic solvents have specific effects on lipase activity; similarly, unique organic chemical syntheses require different organic solvents for appropriate product generation. This characteristic of lipase provides a scientific basis for its use in organic chemical synthesis and for biodiesel production.

One lipase derived from *Rhizomucor miehei* NRRL5282 was found to tolerate organic solvents including low concentrations of methanol, ethanol, propanol, and isopropanol, which had little impact on its enzyme activity (Takó et al., 2017). LipM, a lipase derived from *Pseudomonas moraviensis* M9, is stable in isopropanol, methanol, and ethanol; however, glycerol, polyethylene glycol (PEG)-400, and PEG-600 all inhibit its activity (Yang et al., 2015a). LipR, a lipase derived from *Pseudomonas* sp. R0-14, was inhibited slightly by methanol, while glycerol, dimethyl sulfoxide (DMSO), ethanol and PEG-600 strongly inhibited its activity (Yang et al., 2015b). LipA, a lipase derived from *Pseudomonas protegens* pf-5, was inhibited by chloroform but activated by many other organic solvents, including N-hexane, acetone, glycerol, methanol, tert-butanol and ethanol (Zha et al., 2014).

### Optimum Substrates for Microbial Lipases

The substrate specificity of lipase is determined by the structure of the enzyme molecule, especially the structure of the enzyme activity center, the structure of substrate, and factors influencing substrate binding to the active and other factors affecting enzyme activity. As such, various sources of lipases and lipases with variant structures may display unique substrate specificities.

For example, a lipase derived from *T. lanuginosus*, is actually far more reactive with short-chain pNP-esters substrates (Xin et al., 2017). A lipase derived from *G. stearothermophilus* AH22 has a preference for short-chain methyl ester substrates (i.e., C4; Ekinci et al., 2016). Similarly, a lipase derived from *Geobacillus kaustophilus* DSM 7263^*T*^ showed maximum activity toward short chain fatty acid (C4), and the enzyme activity was decreased with higher carbon number of fatty acids (Özdemir et al., 2021). However, a lipase from *B. ubonensis* SL-4 exhibits high catalytic activity against the medium- to long-chain pNP-esters (i.e., C8-C16; Yang et al., 2016). A lipase derived from *Bacillus* sp. displays a moderate preference for triolein (Sivaramakrishnan and Incharoensakdi, 2015), while lipase LipA derived from *P. protegens* pf-5 performs preferential catalysis with medium pNP-esters, as opposed to short- or long-chain pNP-esters (i.e., C8-C10; Zha et al., 2014). A lipase derived from *P. pasadenensis* displays highest catalytic activity against pNP-esters (C8; Gao et al., 2018), while LipM, a lipase derived from *P. moraviensis* M9, exhibited high catalytic activity with medium- to long-chain pNP-esters substrates (i.e., C8-C16; Yang et al., 2015a).

## The Structure and Catalytic Mechanism of Microbial Lipases

### Structure of Lipase From Microorganisms

Although the molecular weights of microbial lipases can vary from 20 to 60 kDa, they are all members of the α/β-hydrolase fold protein family (Nardini and Dijkstra, 1999; Singh and Mukhopadhyay, 2012). Hydrolases are a class of enzymes whose activity depends on the catalytic triad that includes Ser, His and Asp (Farrokh et al., 2014; Faouzi et al., 2015). In the α/β hydrolases, these three amino acid residues are detected in order as Ser-Asp-His; the Ser residues are all located in a conserved catalytic Gly-X-Ser-X-Gly sequence. This conserved pentapeptide sequence is present in all serine hydrolases and plays a key role with respect to the catalytic activity of this group of lipases (Nardini and Dijkstra, 1999; Schreck and Grunden, 2014).

**FIGURE 2:**
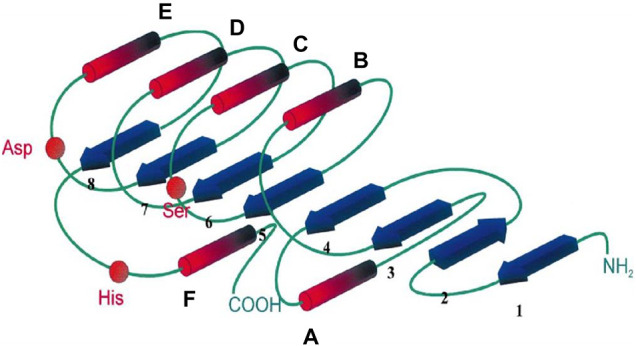
Schematic of the canonical α/β hydrolase fold. The filaments forming the structure in β-pleated sheet (1–8) are indicated by blue arrows and the structures in α-helices **(A–F)** are indicated by red columns. The relative positions of the amino acids of the catalytic triad are indicated by red beads. The amino-terminal region is indicated by NH2 at the beginning of the chain, and by COOH for the carboxy-terminal region for the end of the chain. The figure was reprinted with permission, from Bornscheuer (2002).

All lipases exhibit similar secondary structure; the main differences observed relate to the number and arrangement of α-helices as well as the number and angle of the folds in the β-strands (Schrag and Cygler, 1997). A typical α/β fold is shown in Figure 2.

**FIGURE 3:**
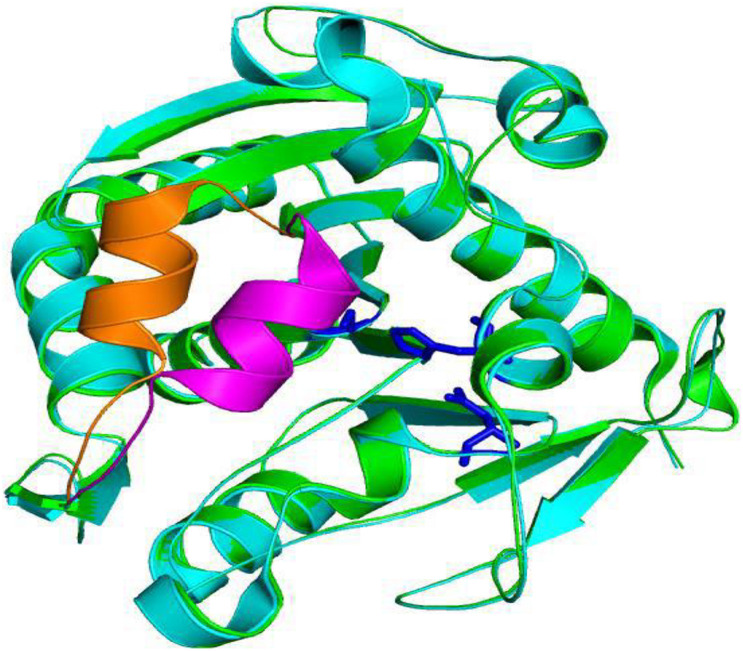
Schematic diagram of superimposition of opening and closing of lipase lid structure of *Thermomyces lanuginosus.* The close lid, open lid, and catalytic triads were highlighted by magenta, orange, and blue colors, respectively (Khan et al., 2017).

Most lipases also contain an unstable domain known as a “lid structure” (Sarmah et al., 2018). When at locales other than oil-water interfaces, the lid structure covers the active site; when located at an oil-water interface, the lid will be opened to permit interactions between the catalytic site and the substrate, thereby facilitating solvent catalyzed reaction (Khan et al., 2017; Rrcm et al., 2020). For example, the structure of lipase from *T. lanuginosus* showed different phenomena when close and open. A comparison of both conformations is shown in Figure 3 (Khan et al., 2017). Another important feature of lipases is the “oxygen anion hole.” Arpigny and Jaeger divided lipases into three types, GGGX, GX and γ, according to the preference of “oxygen anion hole” in catalyzing different substrates (Albayati et al., 2020). In addition, there are special interfacial recognition sites in the structure of lipase, which will change the conformation in the presence of lipid and amphiphilic, and the stability of lipase on the interface (Aloulou et al., 2006).

### Catalytic Mechanisms of Microbial Lipases

**FIGURE 4:**
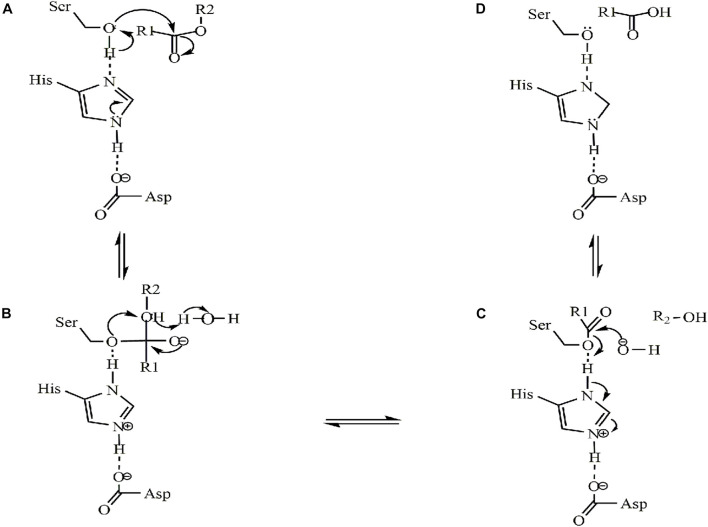
The catalytic mechanism of lipase (Jaeger et al., 1999). **(A)** The enzyme binds to the substrate; **(B)** intermediate tetrahedral structures are formed; **(C)** acyl covalent intermediate complexes are formed; **(D)** deacylation.

The catalytic mechanisms associated with microbial lipases rely on a catalytic triad that is very similar to those identified in serine hydrolases (Schreck and Grunden, 2014). The catalytic mechanism of lipase can be divided into four steps, as shown in Figure 4: (a) at the catalytic triad, the histidine residue removes the hydrogen on the serine hydroxyl group, generating a negatively charged oxygen molecule that can undergo nucleophilic interactions with a positively charged carbonyl carbon, resulting in the formation of a covalent bonds, followed by (b) the lipase electrophilic region (i.e., the oxygen anion hole) is formed, followed by a stable enzyme-substrate tetrahedral transition state, followed by (c) the ester bond is then broken, resulting in the release of the fatty-acid alcohol, formation of acyl covalent intermediate complexes, finally (d) the temporary bonds between the serine and the substrate are then broken, resulting in the release the acyl substrate.

## Heterologous Expression and Molecular Modification of Microbial Lipases

### Heterologous Expression of Microbial Lipases

Heterologous expression is an effective means to promote large-scale industrial production of one or more specific lipase. Heterologous expression systems are associated with significant advantages, including the fact it is typically does not require lipids and other inducers to promote biosynthesis; this reduces the overall complexity of the fermentation process. Common methods used to generate recombinant lipase include bacterial, yeast, baculovirus/insect, mammalian, and filamentous fungal-based expression systems; bacterial, yeast and filamentous fungal-based expression systems are among the most widely used (Valero, 2018).

### Prokaryotic Expression Systems

Heterologous expression in *Escherichia coli* is the most commonly used prokaryotic expression system; this has been facilitated by a deep understanding of the bacterial genome, genetics, physiology, and mechanisms underlying gene expression. *E. coli* grow rapidly in culture and can achieve high-density growth in a short period of time using inexpensive media that is suitable for continuous fermentation. The yield of recombinant protein per unit volume tends to be relatively high, and can represent up to 30% of the secreted protein. Recombinant lipase expressed in *E. coli* can be located in the cytoplasm or secreted to the extracellular environment; it can exist in the form of inclusion bodies or expressed as a fusion protein with a FLAG epitope tag or fluorescent protein in order to avoid intracellular degradation and to simplify the subsequent separation and purification processes (Yin et al., 2007).

However, heterologous expression in *E. coli* is associated with the following disadvantages: (1) when eukaryotic proteins, especially heterologous proteins containing disulfide bonds, are expressed in *E. coli*, inactive cytoplasmic inclusion bodies can be formed due to improper protein folding; (2) Heterologous proteins that are not secreted often do not undergo proper folding and post-translational modification; this can lead to inactivation and degradation of heterologous proteins by the host; (3) the recombinant protein of interest needs to be separated from many background *E. coli* proteins, resulting in the need for complex separation and purification steps; (4) the *E. coli* expression systems involves contamination with lipopolysaccharide (LPS) which is a proinflammatory molecule in mammalian hosts that plays a key role in host-pathogen interaction with the innate immune system. Other commonly used prokaryotic expression systems include those based in *B. subtilis*, *Ralstonia eutropha*, and *Pseudomonas* spp. Compared with the *E. coli* expression system, the *B. subtilis* system facilitates recombinant protein secretion, and has no Gram-negative lipopolysaccharide components; as such, this system is suitable for large-scale high-density fermentation. However, the instability of the bacterial host protease system and associated plasmid loss can result in a decrease in recombinant lipase production. Less is known regarding the expression of recombinant lipases in systems based on *Ralstonia eutropha* or *Pseudomonas* (Valero, 2018).

### Yeast Expression Systems

Similar to bacteria, yeast grow rapidly in culture and are relative simple to use; however, yeast cells promote more complete post-translational modifications that serve to maintain recombinant proteins in native, active state (Madzak et al., 2004). As such, yeast expression systems are used widely in industry, scientific research, and pharmaceutical fields. Common yeast expression systems include those featuring *S. cerevisiae*, *P. pastoris*, *Hansenula*, *Yarrowia lipolytica*, *C. boidinii* expression system, and *Kluyveromyces lactis.*

As a Generally Recognized As Safe microorganism, the genetic background of *S. cerevisiae* has been fully clarified; it is the first eukaryotic organism to be used to promote recombinant gene expression (Boer et al., 2007). However, the expression system of *S. cerevisiae* includes a number of disadvantages, including poor plasmid stability and relatively weak levels of protein secretion; as such, the expression levels of most recombinant proteins are not high. Furthermore, amplification of fermentation process is not straightforward, and there are also problems associated with protein over-glycosylation (Valero, 2018).

By contrast, the *P. pastoris* expression system has the advantage of simplicity, rapid growth rate, and relatively low nutritional requirements. Rather than the aforementioned plasmid-based expression systems, the exogenous genes are integrated into host chromosomes via homologous recombination (Wang et al., 2019), which provides the advantage of genetic stability. Furthermore, exogenous gene expression is strictly regulated under the control of the alcohol oxidase (AOX) promoter in yeast cells. Moreover, compared with the results form prokaryotic expression systems, yeast expression systems produce large amounts of protein that can be released into the extracellular milieu, which facilitates their ultimate separation and purification. Finally, processing mechanisms in yeast cells promote appropriate post-translational modification of recombinant proteins; as such, it is usually easier to generate recombinant proteins with full biological activity.

Expression systems featuring *Yarrowia* and *Hansenula* are also commonly used for recombinant lipase expression; *Yarrowia* have been used to promote overexpression of their own lipase (Janek et al., 2020).

### Filamentous Fungal-Based Expression Systems

The genetic and physiological backgrounds of the filamentous fungi are less well understood than are those used for expression of recombinant proteins in prokaryotic cells and yeast. However, they are clearly multifunctional, and as such, they are in wide used as recombinant expression systems. Filamentous fungi can generally utilize inexpensive raw materials to support growth and to promote efficient expression and secretion of recombinant proteins in a manner that is suitable for large-scale industrial production. The post-translational modification processes within these cells are similar to those of eukaryotes and serve to ensure biological activity of recombinant proteins produced (Su et al., 2011).

### Molecular Modification of Microbial Lipases

Currently, there are only a limited number of microbial lipases that are in use for industrial applications. One of the important means that might be used to broaden the research field and to obtain enzyme preparations with improved physical, chemical, and catalytic properties would be via molecular modification of existing lipases by genetic engineering. The molecular modification strategies used to alter the properties of enzymes have been divided into three categories, including rational design, irrational design and semi-rational design (Rrcm et al., 2020). Appropriate use of rational design strategies presupposes a clear understanding of the three-dimensional structure of the enzyme. In combination with bioinformatics, a large number of simulated docking sites can be selected for modification during the early stages of this procedure. Generally, changes in the protein sequence are introduced via site-directed mutagenesis techniques. By contrast, irrational design does not require any specific understanding of enzyme structure. Introduction of mutations is largely random, as such, this approach is typically combined with high-throughput screening, which can be both time and resource- consuming. Semi-rational design is a combination of these two approaches; this approach typically includes homologous modeling or other means to generate a basic understanding of protein structure to facilitate a semi-rational mutagenesis strategy.

### Irrational Design

Irrational design is also known as directed evolution. In this design process, protein structural and functional information is not needed (Ollis et al., 2016); random mutations are introduced into the target gene via a means that is meant to simulate natural evolution; targeted screening is then conducted so as to identify the protein that meets the specific structural or functional requirements (Albayati et al., 2020). Generally, this process involves two steps, including first, the construction of the mutant library, which needs to be of adequate size and diversity; the second step involves high-throughput screening, which is fast, sensitive, and directional. Druteika et al. used this technique to mutate *Geobacillus* lipase GD-95 from with the goal of improving enzyme activity; it was identified that the specific activity of GD-95RM lipase increased by 1.3 times (2000 U/mg; Druteika et al., 2020).

### Rational Design

The use of a rational design protocol presupposes a knowledge of the three-dimensional structure and function of the protein to be targeted; using this methods, specific changes are introduced based on the understanding of the structure-function relationship. In order to improve the thermostability of r27RCL from *Rhizopus chinensis*, Wang Rui et al. screened four thermostable variants S142A, D217V, Q239F, and S250Y by reasonable design, and then combined together to generate a quadruple-mutation (S142A/D217V/Q239F/S250Y) variant, called m31. m31 showed enhanced thermal stability, and its half-life was prolonged by 41.7 times at 60 °C (Wang et al., 2020).

### Semi-Rational Design

Semi-rational design is a combination of rational design and directed evolution. When designing changes to the DNA sequence based on this technology, some information related to protein structure and function is required. Mutations are introduced to alter the coding sequence at specific points; random and site-specific saturation mutations are carried out simultaneously. Random mutation and screening, followed by site-specific saturation mutation, can be subjected to a scaled-down selection process, according to the needs of the experimental design (Liu et al., 2011). For example, Jiang Hong et al. in order to enhancement of hydrogen peroxide tolerance of lipase LipA from *B. subtilis*, Constructed and screened a minimal Library of *B. subtilis* lipase LiPA composed of 26 mutants based on the known 3D structure information and mass spectrometric analysis. In the mutation library, the C_50_ value and t_1/2_ value of the best mutant variant, LipA-Trp^42^Ala/Met^134^Glu was increased by 9.1-fold and 9.5-fold, respectively (Jiang et al., 2020).

With the development of molecular biology technology, heterologous expression and molecular modification have been widely used in the expression of microbial lipase. Heterologous high efficiency expression of enzyme gene or molecular modification of lipase protein to improve the corresponding enzymatic properties is the most direct and convenient way to obtain a highly active enzyme or improve the corresponding enzymatic properties. In view of the low efficiency of lipase heterologous expression and the need to improve the fermentation level, the appropriate heterologous expression system was selected. Based on heterologous expression systems and to express lipase fitment problem, can use bioinformatics, synthetic biology, systems biology, such as thoughts and technical means, through the prediction and design effective reform way, reconstruct metabolic network, optimize the integration of different fine properties such as strategy, build efficient expression of heterologous lipase system, By optimizing the key technological factors and process regulation in the lipase production process, the optimal control technology of the lipase production process was determined, and the stable and efficient production of exogenous lipase was realized, so that the lipase was developed satisfactorily in industry.

### Applications of Microbial Lipases

Microbial lipase is currently in prominent use in numerous biotechnology and industry applications; the market for these enzymes is expected to expand further over the next 5 years. A great deal of time and effort has been devoted to the development of new technologies that will ultimately serve to increase the utility and application of lipases in a variety of important fields. Among the current industrial applications, microbial lipases are used widely for the production of food, medicine, detergents, paper, biodiesel fuel, textiles, and cosmetics (Newswire, 2018). Major industrial applications of lipases are summarized in Table 2.

**TABLE 2:** Industrial applications of microbial lipases.

Industry	Action	Product or application	References
			
Fats and oils	Transesterification; hydrolysis	Cocoa butter, margarine, fatty acids, glycerol, mono-, and diglycerides	Konkit and Kim, 2016
Dairy foods	Hydrolysis of milk fat, cheese ripening, modification of butter fat	Development of flavoring agents in milk, cheese, and butter	Esteban-Torres et al., 2015; Konkit and Kim, 2016
Bakery foods	Flavor improvement	Shelf-life prolongation	Lamsal and Faubion, 2009
Beverages	Improved aroma	Beverages	Chandra et al., 2020
Food dressings	Quality improvement	Spices and seasonings	Gricajeva et al., 2018
Pharmaceuticals and Medical equipment	Transesterification, hydrolysis	Treatment of tumors, chiral drug resolution, antioxidants, digestive aids and Blood sensor	Dai and Xia, 2005; Herrera-Lopez, 2012; Horchani et al., 2012; Bancerz, 2017; Kumar et al., 2017; Memarpoor-Yazdi et al., 2018
Pulp and paper	Hydrolysis	Paper with improved quality	Demuner et al., 2011; Horchani et al., 2012; Liu et al., 2012; Almeida et al., 2018; Patel et al., 2019
Washing	Hydrolysis	Removal of fats	Niyonzima and More, 2015; Phuah et al., 2015; Kumar et al., 2016; Unni et al., 2016; Holland and Sajic, 2017; Mehta et al., 2017; Saraswat et al., 2017; Abol-Fotouh et al., 2021
Biodiesel	Transesterification, hydrolysis, synthesis	Biodiesel production	Karmee et al., 2015; Tran et al., 2016; Khosla et al., 2017; Tian et al., 2017; Li et al., 2020; Sorte et al., 2020; Talavari et al., 2020
Leather	Hydrolysis	Leather products	Kavitha et al., 2018; Rashid et al., 2018
Cosmetics	Synthesis	Emulsifiers, moisturizers	Gupta et al., 2015; Kim et al., 2015; Ugo et al., 2017; Uppada et al., 2017; Tufiño et al., 2019
Environmental protection	Transesterification, hydrolysis	Environmental detection and repair	Lauprasert et al., 2017; Bucur et al., 2018; Hassan et al., 2018; Ma et al., 2018; Kumar et al., 2020

### Applications of Microbial Lipases in the Food Industry

Traditional processes generally use inorganic acids or metal oxidation to catalyze the hydrolysis or esterification of oil; the reaction conditions are especially harsh, requiring special equipment to carry out the reaction. Furthermore, the reaction cycle is long and involves significant energy consumption, high costs, and the chance of environmental pollution. By contrast, the use of lipase as a biocatalyst can overcome these problems; lipase-mediated reactions are specific and highly efficient. As such, lipase-catalyzed reactions are gradually replacing the traditional methods.

Lipase can also be used to refine oil and by removing free fatty acids; this method may also be used to modify lower-quality oils to obtain a high value-added product (Konkit and Kim, 2016). Lipase can also be used to improve food flavor. In the dairy industry, lipase hydrolyzes milk fat; this could greatly improve the flavor and taste of dairy products and increase their nutritional value (Konkit and Kim, 2016). A lipase purified from *Lactobacillus plantarum* has been used in the synthesis of fermented cheese-based foods (Esteban-Torres et al., 2015). Lipase can also be used to improve the quality of baked goods; it can whiten darker breads, improve the taste and quality of baked foods, and extend their shelf life (Lamsal and Faubion, 2009). Lipase is also commonly used in alcoholic beverage processing to improve flavor and increase product attractiveness (Chandra et al., 2020). Immobilized lipase from *Aspergillus sp.* (Resinase A 2X) may also contribute to the synthesis of perfume; it uses a harmless solvent and the product recovery is simple (Gricajeva et al., 2018). A lipase purified immobilized lipase isolated from *R. miehei* (immobilized on anionic exchange support) have been used as pentyl nonanoate in enhancing fruit aroma (Muniandy et al., 2019).

### Medical Applications of Microbial Lipases

Lipase is a basic enzyme that contributes to the metabolism of fat; it is an important drug target, a biomarker, and has been used to produce drug intermediates such as unsaturated fatty acids. Lipase expression can be regulated to aid in digestion; it has also been used as part of a strategy to treat malignant tumors (Bancerz, 2017).

Lipase can also contribute to the process of chiral drug resolution; selective esterification facilitates separation of the racemic mixture and isolation of a single active enantiomer (Kumar et al., 2017). Dai and Xia (2005) found that the enantioselectivity of lipase was greater than 100 when using *Penicillium* extendus to split the intermediate.-fold. The separation of ibuprofen isomers features the advantages of this technology. *Rhodothermus marinus* DSM 4252 lipase degraded ibuprofen ester in immobilized form to output (S)-enantiopure ibuprofen (Memarpoor-Yazdi et al., 2018).

Lipase-based sensors are used as diagnostic tools to detect triglyceride, cholesterol and phospholipid levels in patient blood samples (Herrera-Lopez, 2012). Similarly, lipase obtained from staphylococcal strains has been used in the manufacture of antioxidants such as ethanol-acetate and eugenyl benzoate (Horchani et al., 2012).

### Application of Microbial Lipase in the Pulp and Paper Industry

Resin is often detected in pulp and paper processing; this may be the result of raw material in the storage tank, resin deposition on the paper stock, and oils from the roller resin binder; this can result paper destruction, downtime, and unstable operation. Resins and oils also present obstacles with respect to machine cleaning and maintenance, and typically result in great inconvenience. Resin deposition will also reduce the efficiency of washing, screening, and purification of pulp. Lipase could be used to remove esters from pulp, thus improving the quality and production capacity (Horchani et al., 2012).

In the papermaking industry, the addition of lipase can remove only the contaminating resins and oils in the paper; this will serve to reduce the breakage and to ensure the paper quality and output (Almeida et al., 2018).

Lipase can also treat anion residue and bitumen deposition in papermaking so as to avoid negative effects on the operation of papermaking equipment (Demuner et al., 2011; Liu et al., 2012). Nippon, one of Japan’s major paper industries, has developed a method to control contamination from wood pitch by effecting their hydrolysis (90%) using *C. rugosa* fungal lipase (Patel et al., 2019).

### Applications of Microbial Lipase in the Detergent Industry

One of the most important commercial applications of lipase is as a detergent additive. Lipases are the second most significant detergent additive after proteases; this accounts for 32% of total lipase sales. According to estimation, one thousand tons of lipases in almost thirteen billion tons of detergent are added every year (Mehta et al., 2017). Lipase added to the detergent can increase the flexibility of the fabric and can enhance decontamination without adding pollution to the environment (Kumar et al., 2016). Lipases added to detergent must be capable of functioning in a strong alkaline environment, at high temperature, and with various surfactants that are typically used in routine detergent preparations.

The lipase source strains commonly used in the detergent industry include those from *B. licheniformis*, *Geobacillus* spp., *Serratia marcescens* DEPTK21, *B. flexus* XJU-1, *Bacillus pumilus* SG2 (Niyonzima and More, 2015), *Staphylococcus arlettae*, *Bacillus cepacia*, *Pseudomonas fluorescens and Candida* spp. (Phuah et al., 2015; Su et al., 2015). There are also some publications reporting that lipase derived from *A. niger* performs well in normal temperature washes and is suitable for use as a detergent additive (Unni et al., 2016). A lipase from *B. subtilis* was resistant to surfactants, oxidizing agents and commercial detergents, suggesting it as a potential candidate for detergent formulation (Saraswat et al., 2017). Others found that *G. stearothermophilus* FMR12 showed high lipolytic activity at 70 °C in pH 9 and has been successfully utilized as detergents (Abol-Fotouh et al., 2021). In 2017, a patent was registered for the use of lipase in washing powder to be used for cold water cleaning (Holland and Sajic, 2017).

### Applications of Microbial Lipases in Biodiesel Production

Biofuels, including biodiesel, have become a renewable alternative to fossil fuels (Shuba and Kifle, 2018; Bilal et al., 2021). The production of biodiesel typically relies on animal fat and vegetable oil as biomass; as such, biodiesel a modern source of clean energy (Filho et al., 2019). Biodiesel is comprised of a fatty-acid monoester that is generated by transesterification of triglycerides from fats (Filho et al., 2019). At present, biodiesel is produced mainly by alkaline catalysis and supercritical fluid catalysis (Mandolesi De Araújo et al., 2013); lipase catalysis has been used in the industrial production of biodiesel (Li et al., 2020; Puna et al., 2020; Talavari et al., 2020). According to the research of Tran et al. (2016) the biodiesel conversion rate using sunflower oil in a packed bed reactor with immobilized *Burkholderia* lipase, they found that a single packed bed reactor resulted in a 67% biodiesel conversion rate. However, when the reaction was carried out in a continuous packed bed reactor, the biodiesel conversion rate was increased to 85% (Tran et al., 2016).

Methanol and edible waste oil were also converted into biodiesel and glycerin via the actions of microbial lipase (Karmee et al., 2015). Such as, *T. lanuginosus* lipase form catalyzed methanolysis of waste oil for the production of biodiesel (Tian et al., 2017). Using the commercial lipase Novozyme^®^ 435 (Made by Novozymes), edible waste oil was used as raw material to produce biodiesel via enzyme-catalyzed regeneration (Sorte et al., 2020). Similarly, Khosla et al. (2017) isolated an extracellular lipase from *Pseudomonas* ISTPL3; lipid production of liposophore ISTD04 was used for transesterification to produce biodiesel fuel (Khosla et al., 2017).

### Other Applications of Microbial Lipases

Microbial lipases have been used as environmentally friendly degreasers in the leather processing industry; lipases were used to replace surfactants in cowhide degreasing. For sheepskin, which contains up to 40% fat, solvents are often used; these can also be replaced by lipases and surfactants (Kavitha et al., 2018). For example, an in-house extracted and purified lipase from *Aspergillus cervinus* was immobilized onto chitosan-alginate (CTS-ALG) beads for sheep skin depilation (Rashid et al., 2018).

Lipases also have the capacity to produce fragrance and thus may be used for the production of cosmetics and perfumes (Gupta et al., 2015; Kim et al., 2015; Ugo et al., 2017; Uppada et al., 2017; Tufiño et al., 2019). Lipase has also been used as a major ingredient in topical creams and in oral medications to aid weight loss through removal fat (Sonne et al., 2015); lipase is also in wide use for the production of curls (Shafqat et al., 2015).

Lipases can be used to detect environmental pollutants, such as pesticides (Bucur et al., 2018; Ma et al., 2018); they have been used frequently for environmental remediation (Kumar et al., 2020). The use of lipase to treat industrial, edible waste oil, and waste water can reduce environmental pollution (Lauprasert et al., 2017; Hassan et al., 2018; Kumar et al., 2020).

## Conclusion and Future Perspectives

Our analysis of the existing literature revealed that microbial lipases are among the most versatile and productive lipases in current use. Microbial lipases function over a wide temperature and pH range, exhibit a remarkable range of substrate diversity and enantioselectivity, and are the first choice for the generation of current and future biocatalysts. Heterologous expression and molecular modification strategies have led to the large-scale application of lipase in industrial production; this has created an important role for microbial lipases in food, pharmaceutical, papermaking, detergent, and biodiesel production, among other fields. Although several microbial strains with lipases capable of functioning at both low and high temperatures have been identified, and efforts have been made toward engineering lipases that exhibit these critical characteristics, issues regarding high production cost and insufficient lipase specificity need to be overcome.

With the increasing application of lipase industrialization, demand for this reagent in the both the papermaking and chemical industries are increasing each day. As such, scientific research in this field is currently focused on ways to identify and to generate new lipases, to expand the search range via the use of bioinformatics, and to employ a wide range of genetic tools and calculations for screening and construction. Among the targets to consider, it would be helpful find ways to improve the properties of existing microbial lipases, including the means to increase activity, productivity, thermal stability, specificity, and antipodal selectivity, as well as to reduce production costs. In addition, methods to improve expansion and optimization of large-scale culture are required; this process requires substantial attention to ensure the economic feasibility of lipase catalytic process. Further research focused on the biochemistry, enzymology, and applications of lipase will play an important role in promoting its future commercial feasibility.

## Author Contributions

TW and KL conceived the study. WY, KL, HL, and YJ wrote the draft of the manuscript. WW and RW critically reviewed the full manuscript content. All authors read and approved the final manuscript.

## Conflict of Interest

The authors declare that the research was conducted in the absence of any commercial or financial relationships that could be construed as a potential conflict of interest.

## Publisher’s Note

All claims expressed in this article are solely those of the authors and do not necessarily represent those of their affiliated organizations, or those of the publisher, the editors and the reviewers. Any product that may be evaluated in this article, or claim that may be made by its manufacturer, is not guaranteed or endorsed by the publisher.

## Abbreviations

β-ME: beta-mercaptoethanol
AOT: di-octyl sulfosuccinate (Aerosol-OT)
AOX: alcohol oxidase
CALB: *Candida antarctica* lipase B
CTAB: cetyl trimethyl ammonium bromide
DEPC: diethylpyrocarbonate
DMSO: dimethyl sulfoxide
DTNB: 5,5’-dithio-bis-[2-nitrobenzoic acid]
DTT: dithiothreitol
EDAC: 1-ethyl-3-(3-dimethyl aminopropyl) carbodiimide
EDTA: ethylene diamine tetraacetic acid
ELISAs: Enzyme-Linked Immunosorbent Assays
LPL: lipoprotein lipase
LPS: lipopolysaccharide
OTAB: octadecyl trimethyl ammonium bromide
pCMBA: para-Chloromethyl benzoic acid
PEG: polyethylene glycol
PMSF: phenyl methane sulfonyl fluoride
RIAs: radioimmunoassays
SDS: sodium dodecyl sulfate
WRK: Woodward’s Reagent K.
